# Exceptional points in a passive strip waveguide

**DOI:** 10.1515/nanoph-2024-0701

**Published:** 2025-03-11

**Authors:** Shamkhal Hasanli, Mehedi Hasan, Hyejin Yoon, Seungyong Lee, Sangsik Kim

**Affiliations:** School of Electrical Engineering, Korea Advanced Institute of Science and Technology, Daejeon 34141, Republic of Korea; Department of Electrical and Computer Engineering, Texas Tech University, Lubbock, TX 79409, USA; Graduate School of Quantum Science and Technology, Korea Advanced Institute of Science and Technology, Daejeon 34141, Republic of Korea; School of Electrical Engineering and Graduate School of Quantum Science and Technology, Korea Advanced Institute of Science and Technology, Daejeon 34141, Republic of Korea

**Keywords:** exceptional points, waveguide, photonic integrated circuit, non-Hermitian photonics

## Abstract

Exceptional points (EPs) in non-Hermitian systems have attracted significant interest due to their unique behaviors, including novel wave propagation and radiation. While EPs have been explored in various photonic systems, their integration into standard photonic platforms can expand their applicability to broader technological domains. In this work, we propose and experimentally demonstrate EPs in an integrated photonic strip waveguide configuration, exhibiting unique deep wave penetration and uniform-intensity radiation profiles. By introducing the second-order grating on one side of the waveguide, forward and backward propagating modes are coupled both directly through second-order coupling and indirectly through first-order coupling via a radiative intermediate mode. To describe the EP behavior in a strip configuration, we introduce modified coupled-mode equations that account for both transverse and longitudinal components. These coupled-mode formulas reveal the formation of EPs in bandgap closure, achieved by numerically optimizing the grating’s duty cycle to manipulate the first- and second-order couplings simultaneously. Experimental observations, consistent with simulations, confirm the EP behavior, with symmetric transmission spectra and constant radiation profiles at the EP wavelength, in contrast to conventional exponential decay observed at detuned wavelengths. These results demonstrate the realization of EPs in a widely applicable strip waveguide configuration, paving the way for advanced EP applications in nonlinear and ultrafast photonics, as well as advanced sensing technologies.

## 1 Introduction

In recent years, exceptional points (EPs) in non-Hermitian systems have emerged as a revolutionary concept in photonics, enabling unique behaviors beyond the realm of traditional Hermitian physics [1]. EPs are unique singularities in the parameter space of non-Hermitian systems, where two or more eigenvalues and their corresponding eigenvectors simultaneously coalesce and become degenerate [1], [2], [3]. Over the last decades, EPs in nanophotonic systems have been engineered to exhibit a range of novel properties [4], such as nonreciprocal light propagation [5], [6], [7], [8], [9], [10], [11], enhanced sensing [12], [13], [14], [15], efficient mode conversion [16], [17], [18], [19], [20], [21], [22], [23], ultralow-threshold lasing [24], [25], [26], [27], [28], [29].

A recent study showed how EPs in periodically modulated media can lead to unconventional phenomena such as deep wave penetration in lossy media and uniform radiation profiles [30]. However, the EP behavior was observed under a planar slab waveguide configuration, where the horizontal dimension of the waveguide is assumed to be infinite [30], [31]. This planar configuration, although effective for studying EPs and their basic properties, poses challenges for practical integration due to its large footprint. Moreover, it limits further applications in nonlinear and ultrafast optics, where higher field confinement is critical for enhancing light–matter interactions [32], [33]. In comparison, the implementation of EPs in a strip waveguide offers substantial advantages, including compact integration, higher field confinement, and compatibility with more complex photonic structures.

In this work, we propose and experimentally demonstrate an integrated photonic strip waveguide configuration that exhibits EPs near the telecom wavelength. We demonstrate this on a silicon nitride (SiN) platform, chosen for its favorable optical properties, including low optical loss, a broad transparency window spanning from visible to mid-infrared wavelengths, high Kerr nonlinearity, and compatibility with CMOS fabrication processes [30], [31], [32], [33]. By introducing a second-order grating on one side of the SiN strip waveguides, we impose a periodic modulation that couples forward and backward waves, both directly and via a radiative intermediary mode. Theoretical analysis reveals the formation of EPs, which we optimize through fully vectorial electromagnetic simulations. Experimental observations, consistent with numerical simulations, reveal a distinct decay-free wave radiation profile at the EP wavelength, while deviations from this wavelength exhibit conventional exponentially decaying radiation.

## 2 Design of exceptional points

**Figure 1:**
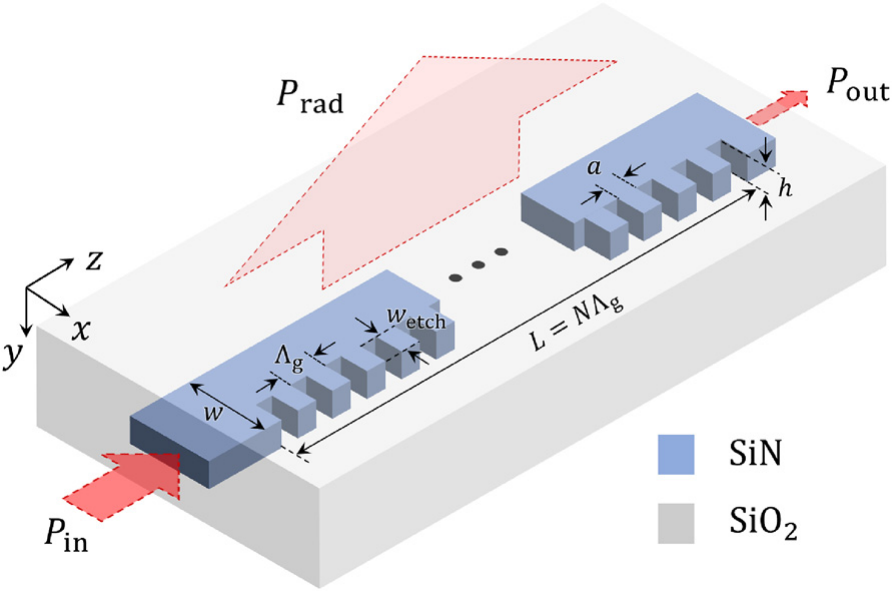
Schematic of the proposed strip waveguide structure for exceptional points (EPs). The design features a second-order grating etched into a SiN strip waveguide cladded by SiO_2_. The periodic grating enables coupling between forward- and backward-propagating modes through direct coupling facilitated by the second-order grating as well as intermediate coupling mediated by the radiative mode. These two couplings can be degenerated by engineering the duty cycle (DC = *a*/Λ_g_) of the grating to realize EPs. Default geometric parameters are: waveguide height *h* = 300 nm, width *w* = 1,200 nm, etch width *w*_etch_ = 400 nm, and grating periodicity Λ_g_ = 990 nm, unless otherwise specified.

A schematic of the design in this work is shown in Figure 1. The blue and gray regions represent SiN and silicon dioxide (SiO_2_), respectively. A uniform grating is patterned on one side of the conventional strip waveguide, which is simple to fabricate through a single etching process. The SiN strip waveguide geometry is designed to support a fundamental TE mode in the near-infrared wavelength range, with a height of *h* = 300 nm and a width of *w* = 1,200 nm. With this geometry, the effective refractive index is approximately *n*_eff_ ≈ 1.568 at wavelengths near 1,550 nm. The grating is designed to support two distinctive couplings: direct coupling between forward- and backward-propagating waves via the second-order grating (*m* = 2), and indirect coupling through a near-vertical radiation mode via the first-order grating (*m* = 1) [30], [34]. To satisfy the phase-matching conditions for the above two couplings, the periodicity Λ_g_ should follow Λ_g_ = *λ*_0_/*n*_eff_, where *n*_eff_ should be the effective value in the grating, not in the strip. For simplicity in device design, we set it as Λ_g_ = 990 nm. The duty cycle (DC) of the grating is defined as the ratio of the high-index region’s length to the total periodicity (DC = *a*/Λ_g_). This DC plays as a key tuning knob for controlling the coupling coefficients to achieve EPs. The grating etching width *w*_etch_ determines the coupling strength between the forward- and backward-propagating modes and also the radiation strength. Here, we fixed this to be *w*_etch_ = 400 nm, which is sufficient for observing the EP characteristics of our devices.

### 2.1 Coupled-mode theory and bandgap engineering for exceptional points

Our device, shown in Figure 1, can be effectively described by the coupled mode theory (CMT) for second-order gratings, with slight modifications to the formulations presented in Refs. [30], [34], [35]. As mentioned, our device supports two distinctive couplings through grating orders *m* = 1 and *m* = 2. The direct coupling between forward- and backward-propagating modes via the second-order grating (*m* = 2) is straightforward. However, the indirect coupling intermediated by a radiation mode at *m* = 1 is not intuitive at a glance. At this grating order, a zero wavevector exists in the system, resulting in an intermediary mode that induces forward-backward coupling. This mode can arise from waves traveling perpendicular to the original propagation direction or from local standing wave resonances and is inherently lossy.

While the CMT for second-order gratings in Refs. [34], [35] effectively describes EPs in 2D gratings [30], it is limited to slab mode, where transverse electromagnetic fields are clearly distinguished (no longitudinal component exists). However, in strip or channel waveguides, as in our configuration, the guided modes are inherently quasi-mode, having both transverse and longitudinal field components. This requires modifications to the conventional CMT to capture the coupling dynamics in a strip waveguide accurately. To address this, we follow the approach described in Refs. [34], [35]. We denote electric and magnetic fields of the forward propagating mode as **e**_*a*_(*x*, *y*) = [*e*_*x*_(*x*, *y*), *e*_*y*_(*x*, *y*), *e*_*z*_(*x*, *y*)] and **h**_*a*_(*x*, *y*) = [*h*_*x*_(*x*, *y*), *h*_*y*_(*x*, *y*), *h*_*z*_(*x*, *y*)], respectively. Similarly, the corresponding fields of the backward propagating mode are denoted as **e**_*b*_(*x*, *y*) = [*e*_*x*_(*x*, *y*), *e*_*y*_(*x*, *y*), −*e*_*z*_(*x*, *y*)] and **h**_*b*_(*x*, *y*) = [−*h*_*x*_(*x*, *y*), −*h*_*y*_(*x*, *y*), *h*_*z*_(*x*, *y*)]. While the spatial field distributions are identical for both propagation directions–since they represent the same mode–the longitudinal field component *e*_*z*_(*x*, *y*) exhibits a sign reversal due to the opposite propagation direction. This sign reversal also applies to *h*_*x*_(*x*, *y*) and *h*_*y*_(*x*, *y*). Assuming that higher-order diffractions rapidly decay and do not carry energy, we can write total electric and magnetic fields in the perturbed waveguide as a superposition of the forward and backward propagating modes along with the radiative mode [35]:

(1a) $$\begin{array}{ll} E\left(x,y,z\right) & =A\left(z\right){\text{e}}^{\text{i}k_{\text{g}}z}\;e_{a}\left(x,y\right)+B\left(z\right){\text{e}}^{-\text{i}k_{\text{g}}z}\;e_{b}\left(x,y\right)\\ & \;+E_{\text{rad}}\left(x,y,z\right), \end{array}$$

(1b) $$\begin{array}{ll} H\left(x,y,z\right) & =A\left(z\right){\text{e}}^{\text{i}k_{\text{g}}z}\;h_{a}\left(x,y\right)+B\left(z\right){\text{e}}^{-\text{i}k_{\text{g}}z}\;h_{b}\left(x,y\right)\\ & \;+H_{\text{rad}}\left(x,y,z\right), \end{array}$$

where, *A*(*z*) and *B*(*z*) represent the modal amplitudes of the forward and backward propagating modes, respectively, described by *A*(*z*) exp(*iω*_b_/*v*_g_ × *z*) and *B*(*z*) exp(−*iω*_b_/*v*_g_ × *z*), along the *z* axis. The Bragg center frequency *ω*_b_ is defined as *ω*_b_ = 2*πv*_g_/Λ_g_, where *v*_g_ is the group velocity in the medium. The *k*_g_ = 2*π*/Λ_g_ represents the grating vector. The terms **E**_rad_(*x*, *y*, *z*) and **H**_rad_(*x*, *y*, *z*) represent the radiative electric and magnetic field components, respectively, playing a key role in mediating indirect coupling between forward and backward propagating modes through first-order diffraction.

After substituting Eq. (1) in Maxwell’s equations and rewriting the terms (see Supplementary Information), the modified CMT for a second-order grating in a strip waveguide can be represented as follows:

(2) $$\begin{array}{ll} \frac{\text{d}}{dz}\left[\begin{array}{c} A\\ B \end{array}\right] & =\left[\begin{array}{cc} \frac{i\Delta \omega }{v_{g}} & ih_{2}\\ -ih_{2} & -\frac{i\Delta \omega }{v_{g}} \end{array}\right]\left[\begin{array}{c} A\\ B \end{array}\right]\\ & \;+h_{1t}\left(A+B\right)\left[\begin{array}{c} -1\\ 1 \end{array}\right]\\ & \;+h_{1z}\left(A-B\right)\left[\begin{array}{c} 1\\ 1 \end{array}\right], \end{array}$$

where Δ*ω* is the detuning from Bragg center frequency *ω*_b_. The intrinsic material losses are neglected in these calculations. In the matrix, the off-diagonal element *h*_2_ corresponds to the coupling coefficient from second-order diffraction, which characterizes the direct coupling. On the other hand, *h*_1*t*_ and *h*_1*z*_ describe the coupling via the radiative mode. Notice that, unlike the CMT equation in Ref. [30], here we introduce two terms to describe the distinct radiative couplings, where *h*_1*t*_ and *h*_1*z*_ correspond to the indirect coupling coefficients associated with transverse (*x*- and *y*-directions) and longitudinal (*z*-direction) fields, respectively.

To qualitatively understand this, we should examine how the stationary radiative mode facilitates coupling. The radiative intermediate mode can generally coupled to both stationary combinations of the two traveling waves, defined by the amplitudes (*A* + *B*) and (*A* − *B*). In Ref. [30], coupling to (*A* − *B*) is prohibited by symmetry, resulting in a bound state in the continuum (BIC) mode [36], [37], [38]. Here, however, coupling to the (*A* − *B*) combination occurs because the longitudinal electric field component *E*_*z*_ is present in the strip waveguide and exhibits a sign inversion for the back-propagating mode. This makes (*A* − *B*) the symmetric combination for *E*_*z*_, while (*A* + *B*) is anti-symmetric and thus non-radiative. This phenomenon will be illustrated later with simulation results.

For an infinite periodic medium, the complex eigenfrequencies Δ*ω*_1,2_ can be obtained by directly solving the differential equation [34]:

(3) $$\frac{\Delta \omega_{1,2}}{v_{\text{g}}}=-i\left(h_{1t}-h_{1z}\right)\pm \sqrt{{\left(h_{2}+ih_{1}\right)}^{2}+k^{2}}.$$

Here, *h*_2_ can be set to a real value by aligning the origin *z* = 0 with the symmetry plane [35]. For simplicity, we substitute *h*_1_ = *h*_1*t*_ + *h*_1*z*_. For the specific case of Re(*h*_2_ + *ih*_1_) = 0, the bandgap disappears at *k* = 0, leading to two degenerate modes at *k*_EP_ = Re(*h*_1_), which correspond to EPs. This condition is consistent with the slab grating configurations reported in Ref. [30].

Note that, in realizing EPs, it is critical to engineer the coupling ratio between the direct coupling *h*_2_ and indirect coupling *h*_1_ to satisfy the condition Re(*h*_2_ + *ih*_1_) = 0. This can be achieved by tailoring the DC of the second-order grating [30]. To this end, we conducted bandgap simulations on our SiN EP waveguide using the 3D finite element method (FEM).

**Figure 2:**
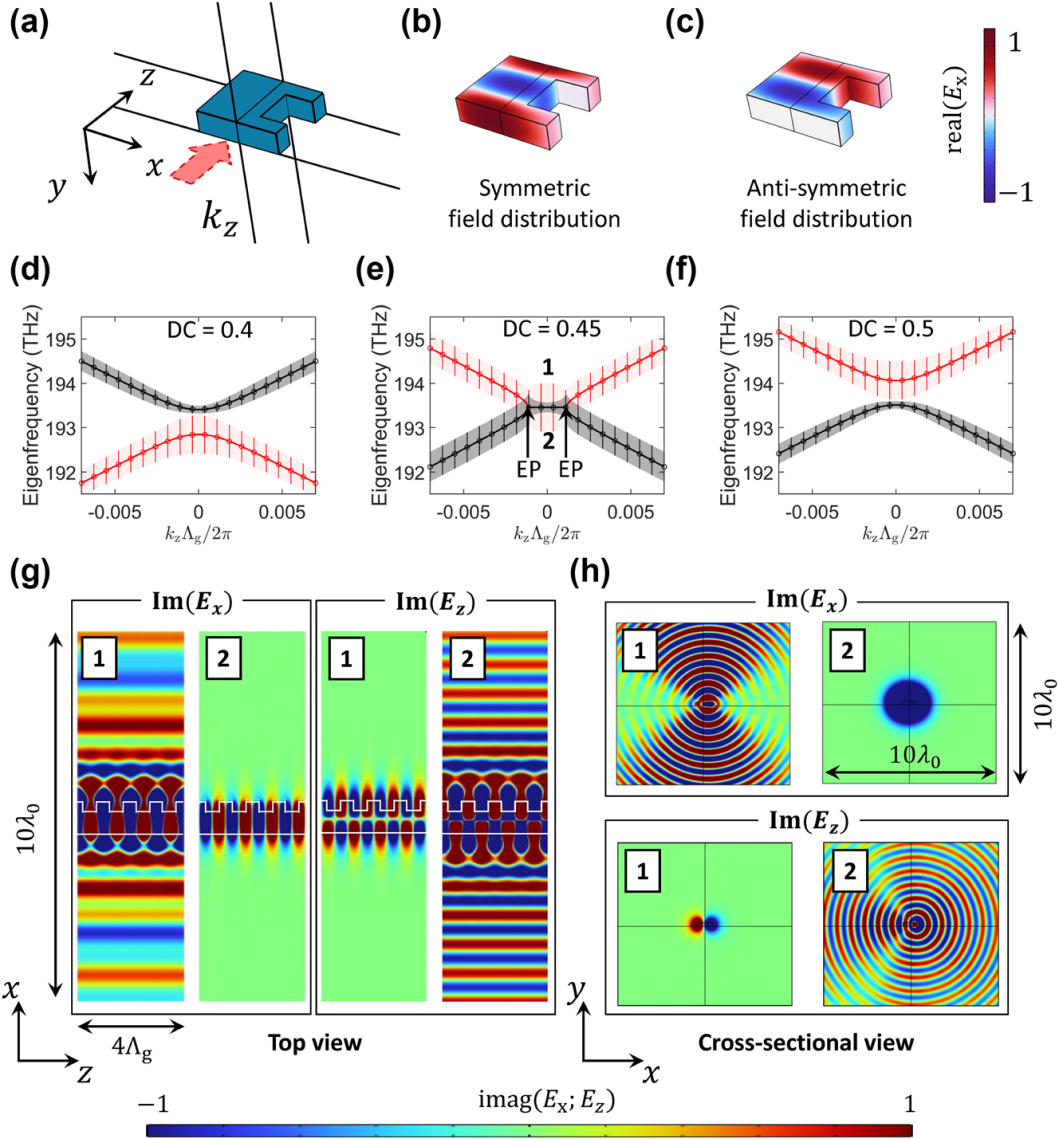
Bandgap engineering of the second-order grating to realize EPs. (a) Perspective 3D schematic of the grating unit cell used in bandgap simulations. (b, c) Field profiles (real *E*_*X*_) of the two degenerate eigenmodes in the EP condition: (b) mode 1 with a symmetric field pattern and (c) mode 2 with an anti-symmetric pattern. The symmetric and anti-symmetric patterns are reversed for the *E*_*z*_ field. (d–f) Band diagrams for various duty cycles (DCs): (d) 0.4, (e) 0.45, and (f) 0.5. Scatter plot markers represent the real part of eigenfrequencies obtained via the finite element method (FEM) simulations, and solid curves correspond to the real part of the eigenfrequencies from coupled mode theory (CMT) calculations. Vertical lines and shaded regions indicate the imaginary eigenfrequencies via FEM and CMT, respectively, representing the radiation loss. (g, h) Field profiles (imaginary *E*_*X*_ and *E*_*Z*_) of the eigenmodes 1 and 2 marked in (e): (g) top view (*xz*-plane) and (h) cross-sectional view (*xy*-plane). In mode 1, radiation arises from the symmetric *E*_*X*_ component, while the anti-symmetric *E*_*Z*_ remains non-radiative. Conversely, in mode 2, the anti-symmetric *E*_*X*_ field does not contribute to radiation, whereas the symmetric *E*_*Z*_ component shows radiative behavior.

Figure 2(a) illustrates the unit cell of the proposed EP waveguide. The waveguide consists of a 300 nm-thick SiN core (*w* = 1,200 nm and *w*_etch_ = 400 nm) cladded by the SiO_2_. Floquet periodic boundary conditions are applied at opposite boundaries of the unit cell along the propagation direction. The real part of *E*_*x*_ for the two degenerate eigenmodes are plotted in Figure 2(b) and (c), representing the degenerate mode 1 and mode 2 indicated in Figure 2(e), respectively. Note that, for the given unit cell period, *E*_*x*_ of mode 1 in Figure 2(b) exhibits a symmetric field profile, while *E*_*x*_ of mode 2 in Figure 2(c) exhibits an anti-symmetric profile.

Figure 2(d)–(f) are the simulated bandgap diagrams for DCs of (d) 0.4, (e) 0.45, and (f) 0.5, respectively. Scatter marks represent the real parts of the FEM-simulated eigenfrequencies, while vertical lines indicate their imaginary parts. Solid lines and shaded regions show the corresponding results obtained by CMT in Eq. (2), demonstrating excellent agreement between FEM simulations and CMT analysis. The coupling coefficients *h*_1_ and *h*_2_ are the key parameters that can be obtained by modal profiles, Fourier coefficients, and Green functions [35]. More detailed calculations are presented in Supplementary Information Section 1. At DC = 0.45 (Figure 2(e)), the bandgap closes, indicating the onset of EP conditions (thus, DC_EP_ = 0.45). However, deviations from DC_EP_ reopens the bandgap, breaking the degeneracy, as shown in Figure 2(d) and (f). Notably, the band-flip phenomenon [39] is observed, where the upper and lower band modes exchange positions. This behavior highlights the critical role of DC tuning in achieving EPs.

Figure 2(g) and (h) show the imaginary parts of the electric fields *E*_*x*_ and *E*_*z*_ for the EP waveguide. Figure 2(g) shows the top view (*xz*-plane), while Figure 2(h) corresponds to the cross-sectional view (*xy*-plane). The plots labeled 1 and 2 correspond to the red and black bands marked in Figure 2(e) at *k* = 0. Mode 1 exhibits strong radiation in the *E*_*x*_ field, while the *E*_*z*_ field remains non-radiative. In contrast, mode 2 shows no radiation in the *E*_*x*_ field but exhibits clear radiation in the *E*_*z*_ field. This reversal in the radiative characteristics of transverse (*E*_*x*_) and longitudinal (*E*_*z*_) field components aligns with the CMT discussed earlier, where anti-symmetric field profiles suppress radiation, while symmetric profiles contribute radiative behavior. Specifically, mode 1 is characterized by a symmetric *E*_*x*_ and an anti-symmetric *E*_*z*_ field pattern in each unit cell, corresponding to (*A* = *B*), whereas mode 2 exhibits an anti-symmetric *E*_*x*_ and a symmetric *E*_*z*_ field, associated with (*A* = −*B*). As a result, both modes 1 and 2 exhibit some degree of radiation, originating from either the transverse or longitudinal components, which diminishes the BIC mode observed in a slab EP configuration [30]. The radial patterns in Figure 2(h) exhibit strong vertical radiation with minimal horizontal radiation. This suggests that the radiation field profile can be effectively observed using a top-view microscope image, which will be presented later in this manuscript. The field profiles of *E*_*x*_ and *E*_*z*_ outside the bandgap closing region, along with the *E*_*y*_ field distributions, are presented in Supplementary Information Section 2.

### 2.2 Spectral response and flat radiation profile at exceptional points

After confirming the bandgap closing in EP setting through FEM simulations and CMT analysis, we now examine the spectral characteristics and radiation profiles of the EP waveguide with a finite grating length *L*. First, by solving the CMT equation in Eq. (2) with boundary conditions *A*(*z* = 0) = 1 and *B*(*z* = *L*) = 0 under the EP condition [Re(*h*_2_ + *ih*_1_) = 0], both *A*(*z*) and *B*(*z*) become linear functions, as follows [30]:

(4a) $$A\left(z\right)=\frac{1-\text{Re}\left(h_{1}\right)\left(z-L\right)}{1+\text{Re}\left(h_{1}\right)L},$$

(4b) $$B\left(z\right)=\frac{\text{Re}\left(h_{1}\right)\left(z-L\right)}{1+\text{Re}\left(h_{1}\right)L}.$$

The radiation power *P*(*z*) can then be calculated as $P\left(z\right)\propto |\frac{\text{d}}{dz}\left(|A\left(z\right){|}^{2}-|B\left(z\right){|}^{2}\right)|$ , which simplifies to:

(5) $$P\left(z\right)\propto \frac{2\text{Re}\left(h_{1}\right)}{{\left(1+\text{Re}\left(h_{1}\right)L\right)}^{2}}.$$

Notably, under the EP condition, the radiation power *P*(*z*) is constant across the grating, independent of the grating coordinate *z*. This constant power depends only on the grating length *L* and coupling coefficient *h*_1_. This behavior represents a unique radiation regime associated with EPs, in contrast to the exponentially decaying radiation observed in conventional gratings.

**Figure 3:**
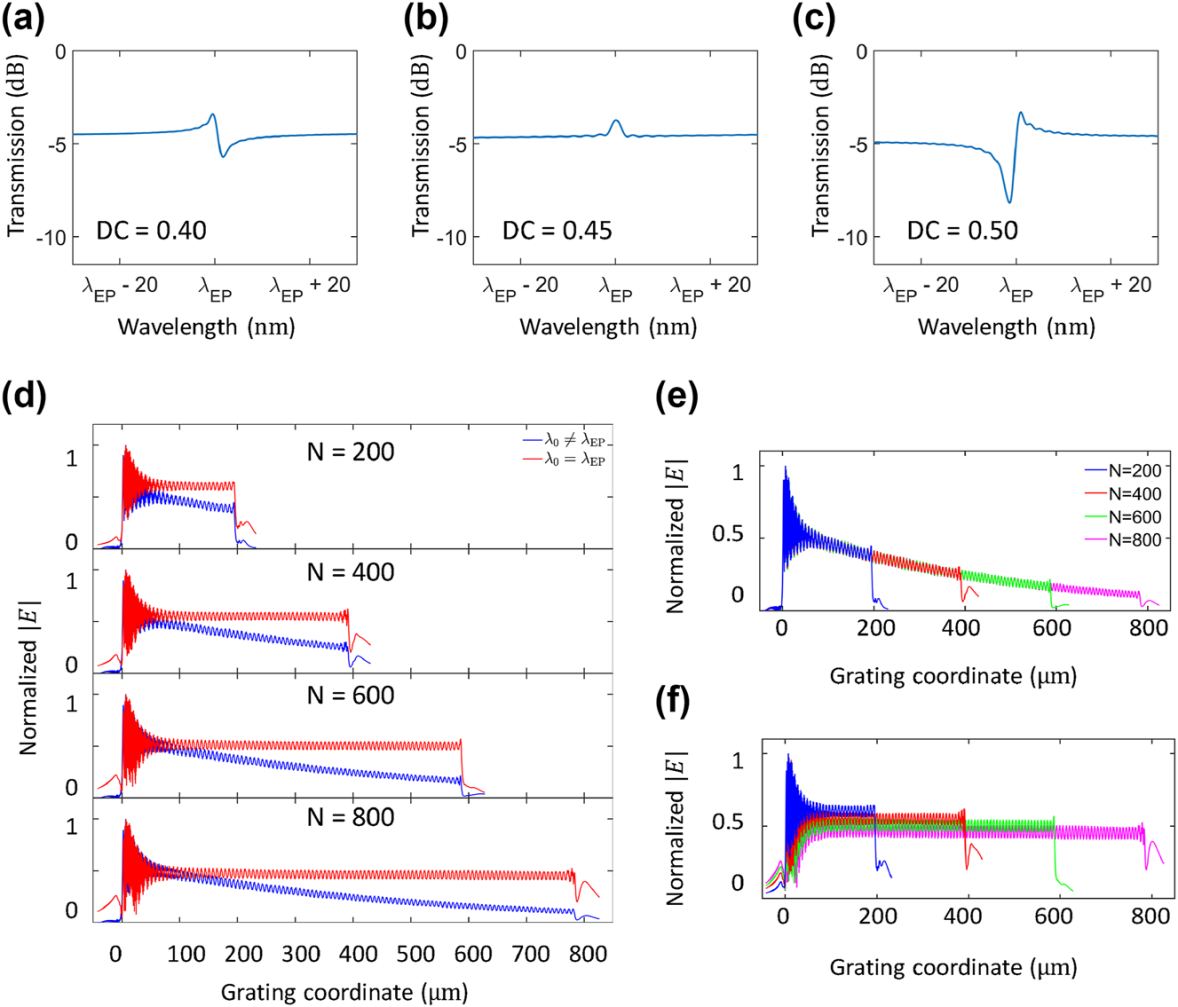
Spectral responses and radiation field profiles of the EP and non-EP gratings. (a–c) Simulated transmission spectra for devices with different DCs: (a) DC = 0.4, (b) DC = 0.45, and (c) DC = 0.5. The spectra exhibit an asymmetric Fano lineshape for DC = 0.4 and 0.5 (non-EP), in contrast to the symmetric response at DC = 0.45, which corresponds to the EP condition. (d) Normalized radiation field profiles |*E*| for the EP device in (b) along the grating for lengths *N* = 200, 400, 600, and 800. Profiles are shown for cases where *λ*_0_ ≠ *λ*_EP_ (blue) and *λ*_0_ = *λ*_EP_ (red). (e, f) Replotted normalized |*E*| along the gratings: (e) for *λ*_0_ ≠ *λ*_EP_, the radiation exhibits conventional exponential decay. (f) For *λ*_0_ = *λ*_EP_, the radiation profile is decay-free, showing uniform intensity across the entire grating, regardless of its length.

To confirm this non-trivial constant-intensity radiation, we performed finite-difference time-domain (FDTD) simulations on the same structure. To accommodate a long grating with up to the number of periods *N* = 800, we employed 2.5D FDTD simulations. Figure 3(a)–(c) show the simulated transmission spectra of our EP waveguide, where the fundamental TE mode was excited for different DCs: (a) 0.4, (b) 0.45, and (c) 0.5. The number of grating periods was set to *N* = 400, making the entire grating length of *L* = Λ_g_ *N* = 396 μm, where Λ_g_ = 990 nm. With these parameters, the EP wavelength appears at *λ*_EP_ ≈ 1,545 nm. At DC = 0.40 (Figure 3(a)), the transmission spectrum exhibits an asymmetric Fano lineshape, characterized by a sharp peak and dip near *λ*_EP_. At DC = 0.50 (Figure 3(c)), the spectrum shows a similar but inverted Fano lineshape around the same wavelength. These nontraditional transmission peaks arise from the interplay between direct coupling and additional indirect radiative coupling and more detailed mathematical descriptions are provided in Section 3 of Supplementary Information. In contrast, at DC = 0.45 (Figure 3(b)), the transmission spectrum becomes a symmetric lineshape, indicating the realization of EPs [30]. At this specific DC, the resonant modes coalesce, resulting in a balanced transmission profile with minimal interference and a pure, symmetric resonance. This symmetric profile reflects the unique conditions of the EP, where the system exhibits degeneracy in both resonance frequency and linewidth.

To further investigate the radiation profiles, we recorded the radiation intensity 10 μm away from the waveguide center. Figure 3(d) shows the normalized electric field profile |*E*| of a radiated free-space beam at EP wavelength (*λ*_0_ = *λ*_EP_, red) and a detuned wavelength (*λ*_0_ ≠ *λ*_EP_, blue) across grating lengths corresponding to *N* = 200, 400, 600, and 800 (from top to bottom). For the EP-tuned wavelength, the beam intensity remains constant across the entire grating length, independent of the grating length, while for the detuned wavelength, the radiation exhibits a conventional exponential decay profile. In every radiation field, small oscillations appear, which might be due to Fabry-Perot-type resonances caused by slight reflections at each interface between the strip waveguide and the grating region.

The distinct wave propagation regimes are further highlighted by replotting Figure 3(d) for the detuned wavelength (Figure 3(e)) and the EP wavelength (Figure 3(f)). Figure 3(e) and (f) clearly compare the difference between the conventional regime (detuned from EP) and the deep wave penetration regime at the EP wavelength. For the detuned wavelength *λ*_0_ ≠ *λ*_EP_, the radiation profile exhibits exponential decay. This arises from the uniform grating, where an identical scattering rate is applied at each grating period. That is, once the grating is extended long enough, the input wave cannot penetrate deeply into the grating. In contrast, at the EP-tuned wavelength *λ*_0_ = *λ*_EP_, the radiation profile is decay-free and remains constant, regardless of the grating length. This unique characteristic of EPs enables deep wave penetration with uniform radiation across the entire grating. The radiated power decreases as the grating length *L* increases, consistent with Eq. (5), which predicts that radiation power is inversely proportional to the square of the grating length. These results strongly align with predictions from CMT, further validating the unique radiation characteristics associated with EPs.

## 3 Experimental results

**Figure 4:**
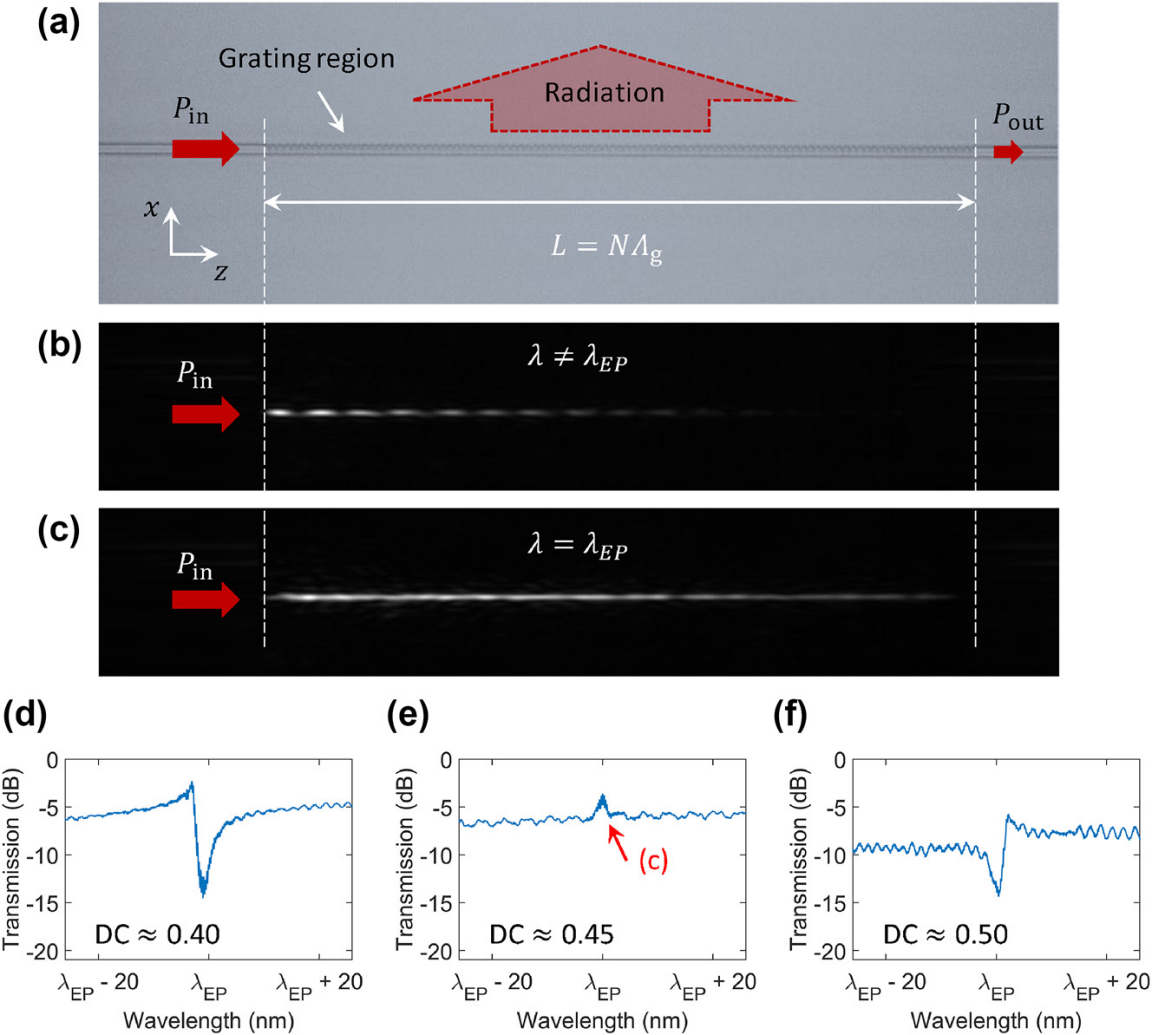
Experimental demonstration of the EP waveguide. (a) Optical microscope image of a fabricated device, showing the input/output ports and radiation. (b, c) Infrared camera images of the EP waveguide (DC ≈ 0.45), capturing the radiation profiles with optical input from the left: (b) *λ*_0_ ≠ *λ*_EP_ and (c) *λ*_0_ = *λ*_EP_. At *λ*_0_ = *λ*_EP_, deep wave penetration through the devices and relatively flat radiation are observed, whereas *λ*_0_ ≠ *λ*_EP_ results in conventional exponential decay. (d–f) Measured transmission spectra for devices with DCs of approximately (d) 0.4, (e) 0.45, and (f) 0.5. The symmetric spectrum at DC ≈ 0.45 confirms the EP condition, while the asymmetric Fano profiles at DC ≈ 0.4 and 0.5 indicate detuned configurations.

For experimental validation of the EP waveguide, uniform grating waveguides with the dimensions specified in Figure 1(a) were fabricated, with the exception of the grating periodicity. In this experiment, a grating period of Λ_g_ = 980 nm was fabricated. A silicon wafer with a thermally grown 10 μm-thick oxide box layer was used as a substrate. A 300 nm thick SiN film was deposited via low-pressure chemical vapor deposition (LPCVD). The grating and edge couplers were patterned using electron-beam lithography, followed by a reactive ion etching process and the deposition of a 1 μm-thick SiO_2_ top cladding in an LPCVD furnace. The number of grating periods was set to *N* = 400, with DCs of approximately 0.4, 0.45, and 0.5. Considering the fabrication tolerances of ±5 nm, the potential DC variations are estimated to be approximately ±0.01. A microscopic image of one of the fabricated devices is shown in Figure 4(a), while Figure 4(b) and (c) show the infrared camera images capturing the radiative field profiles for the same device at different wavelengths.

To characterize the devices, a tunable laser source (Keysight 81608A) light was coupled to the fundamental TE mode through tapered fiber-to-chip edge couplers. A polarization controller placed after the laser source defined the input polarization state. For light injection into the edge coupler, lensed fibers with a mode field diameter of ≈5 μm were used. The output light signals were subsequently directed to the photodetector (Keysight 81635A) through the other port of the chip. Simultaneously, infrared images were captured from the top (WiDy SenS 320), focusing slightly off the waveguide surface to take the radiation beam profile. Figure 4(d)–(f) plot the normalized transmission spectra for devices with DCs of approximately 0.4, 0.45, and 0.5, respectively. For DCs deviating from DC_EP_, the spectra exhibit an asymmetric Fano lineshape, consistent with the simulated results in Figure 3(a) and (c). This asymmetric profile arises from interference between a discrete resonance mode and a continuum of states, a hallmark of Fano resonance behavior [40]. The two opposite Fano lineshapes are a strong indicator for identifying an EP setting, suggesting that the EPs are likely to exist between the two DCs. Indeed, at DC ≈ 0.45, corresponding to DC = DC_EP_, the Fano asymmetry vanishes, and the transmission spectrum becomes symmetric. This symmetric resonance profile indicates the coalescence of the system’s resonant modes at the EP, resulting in balanced transmission with minimal interference.

Figure 4(b) and (c) show the captured radiation profiles at *λ*_0_ ≠ *λ*_EP_ ( ≈ 1,550 nm) and *λ*_0_ = *λ*_EP_ ( ≈ 1,477 nm), respectively, for the EP device with DC_EP_ ≈ 0.45. At the detuned wavelength from *λ*_EP_, the radiation beam exhibits typical exponential decay, as shown in Figure 4(b). In contrast, at *λ*_0_ = *λ*_EP_, the radiation profile extends significantly, approximating a uniform intensity distribution, as shown in Figure 4(c). For identifying the *λ*_EP_ wavelength, we referred to the spectral response in Figure 4(e), which measured *λ*_EP_ ≈ 1,477 nm. We assume this *λ*_EP_ difference between the simulations and experiments is due to the discrepancy in periodicity and further fabrication imperfections. We also annealed the chip to lower the propagation loss, which might reduce the waveguide height as well (we observed color difference in the waveguide under the same microscope, which is a strong indicator of thickness variations).

## 4 Conclusions

In this study, we proposed and experimentally demonstrated EPs on a SiN strip waveguide, showing the unconventional deep-wave penetration and uniform-intensity radiation profile, along with EP-indicating transmission characteristics. The one-side patterned second-order grating facilitates couplings between forward and backward propagating waves, both via direct second-order coupling and indirect first-order coupling through an intermediate radiation mode. We introduced a modified CMT for second-order grating that incorporates both transverse and longitudinal field components, improving the theoretical description of coupling dynamics in strip waveguide-based second-order grating configurations. Compared to slab-based models, which account only for transverse components, our model reveals that the BIC mode is not supported even at the bandgap closure with EPs, due to the additional radiative contribution from the longitudinal field component. Theoretical analysis using CMT revealed the existence of EP bandgap when the first- and second-order coupling coefficients *h*_1_ and *h*_2_ satisfy the condition Re(*h*_2_ + *ih*_1_) = 0. This condition can be achieved by adjusting the DC, which controls the ratio of *h*_1_ to *h*_2_. FEM simulations confirmed the existence of EPs and identified 0.45 as the DC_EP_ that balances *h*_1_ and *h*_2_ to realize the EP band. The spectral characteristics and radiation profiles of the EP waveguide were examined through both FDTD numerical simulations and experimental measurements, which consistently demonstrated symmetric resonance lineshapes at DC_EP_ and constant-intensity radiation profiles at the EP wavelength (*λ*_0_ = *λ*_EP_), in contrast to the asymmetric Fano lineshapes and exponential decay observed at detuned conditions.

The implementation of EPs in SiN strip waveguides offers significant potential for advancing integrated photonic applications, particularly by leveraging the high confinement of strip waveguides to enhance nonlinear processes [41]. The unique and relatively underexplored roles of EPs in nonlinear and ultrafast optics could be explored through the enhanced optical density of states and EP properties, advancing processes such as frequency conversion, optical parametric processes, and ultrafast light modulation. Future research could explore these nonlinear effects in EP-enabled SiN devices or in other material platforms, particularly in configurations like coupled waveguides and microresonators. Furthermore, EPs hold promise for other applications, including enhanced sensitivity in optical sensing [13] and optical mode conversion via encircling EPs [17], [18], [21].

In conclusion, this work demonstrates the feasibility of engineering EPs within SiN strip waveguides, contributing to the advancement of non-Hermitian photonics. While we focused on the implementation of EPs on a SiN platform, the method outlined in this study can be extended to other material platforms and wavelength regimes. By combining the unique properties of EPs with the compactness and high nonlinearity of the hosting material platform (e.g., SiN, as in this study), this work establishes a foundation for next-generation photonic devices, offering transformative potential for integrated photonics utilizing EPs.

## Supplementary Material

Supplementary Material Details
