# Supplementary material for: Exceptional points in a passive strip waveguide
Source: Nanophotonics. 2025 Mar 11;14(8):1301–9. doi: 10.1515/nanoph-2024-0701 (PMC12019939; doi:10.1515/nanoph-2024-0701)
Supplement: Supplementary file 1 — Supplementary Material Details [file j_nanoph-2024-0701_suppl_001.docx]

**Supplementary information for**

**Exceptional points in a passive strip waveguide**

Shamkhal Hasanli^1^, Mehedi Hasan^2^, Hyejin Yoon^3^,
Seungyong Lee^1^, and Sangsik Kim^1,3^*

^1^ School of Electrical Engineering, Korea Advanced Institute of Science and Technology, Daejeon 34141, Republic of Korea

^2^ Department of Electrical and Computer Engineering, Texas Tech University, Lubbock, Texas 79409, USA

^3^ Graduate School of Quantum Science and Technology, Korea Advanced Institute of Science and Technology, Daejeon 34141, Republic of Korea

*Correspondence to be addressed to: [sangsik.kim@kaist.ac.kr](mailto:sangsik.kim@kaist.ac.kr)

1. **CMT of strip waveguide-based second-order gratings**

To describe the couplings due to first and second order diffraction in strip waveguides, we follow the approach in [1,2]. First, we denote $\mathbf{e}_{\mathbf{a}}\left( x,y \right)= \left[ e_{x}\left( x,y \right)\mathbf{,}e_{y}\left( x,y \right),e_{z}\left( x,y \right) \right]$ and $\mathbf{h}_{\mathbf{a}}\left( x,y \right)=\left[ h_{x}\left( x,y \right)\mathbf{,}h_{y}\left( x,y \right),h_{z}\left( x,y \right) \right]$ as the electric and magnetic fields of the forward propagating fundamental mode in unperturbed waveguide. Similarly, the corresponding fields of the backward propagating mode are represented as $\mathbf{e}_{\mathbf{b}}\left( x,y \right)= \left[ e_{x}\left( x,y \right)\mathbf{,}e_{y}\left( x,y \right),{-e}_{z}\left( x,y \right) \right]$ and $\mathbf{h}_{\mathbf{b}}\left( x,y \right)=\left[ -h_{x}\left( x,y \right)\mathbf{, -}h_{y}\left( x,y \right),h_{z}\left( x,y \right) \right]$. Moving forward with the idea that higher order diffractions generate rapidly decaying fields which carry no energy, we can approximate total field in the perturbed waveguide as summation of the fields of forward and backward propagating modes and radiation mode [1]:

$$\begin{aligned} \mathbf{E}\left( x,y,z \right)={A\left( z \right)e^{ik_{g}z}\mathbf{e}}_{\mathbf{a}}\left( x,y \right)\boldsymbol{+}{B\left( z \right)e^{-ik_{g}z}\mathbf{e}}_{\mathbf{b}}\left( x,y \right)\boldsymbol{+}\mathbf{E}_{\mathbf{rad}}\left( x,y,z \right)\boldsymbol{\#}\left( 1a \right) \end{aligned}$$

$$\begin{aligned} \mathbf{H}\left( x,y,z \right)={A\left( z \right)e^{ik_{g}z}\mathbf{h}}_{\mathbf{a}}\left( x,y \right)\boldsymbol{+}{B\left( z \right)e^{-ik_{g}z}\mathbf{h}}_{\mathbf{b}}\left( x,y \right)\boldsymbol{+}\mathbf{H}_{\mathbf{rad}}\left( x,y,z \right)\boldsymbol{\#}\left( 1b \right) \end{aligned}$$

where $\mathbf{E}_{\mathbf{rad}}\left( x,y,z \right)$ and $\mathbf{H}_{\mathbf{rad}}\left( x,y,z \right)$ are electric and magnetic fields present due to radiation. $\mathbf{E}\left( x,y,z \right)$and $\mathbf{H}\left( x,y,z \right)$ are subject to Maxwell’s equations:

$$\begin{aligned} \boldsymbol{\nabla}\boldsymbol{\times}\mathbf{E}\left( x,y,z \right)\boldsymbol{=}i\omega\mu_{0}\mathbf{H}\left( x,y,z \right)\boldsymbol{\#}\left( 2a \right) \end{aligned}$$

$$\begin{aligned} \boldsymbol{\nabla}\boldsymbol{\times}\mathbf{H}\left( x,y,z \right)=-i\omega\epsilon_{0}n^{2}\left( x,y,z \right)\mathbf{E}\left( x,y,z \right)\boldsymbol{\#}\left( 2b \right) \end{aligned}$$

where $n^{2}\left( x,y,z \right)$ is periodically changing refractive index and can be written in terms of Fourier coefficients as:

$$\begin{aligned} n^{2}\left( x,y,z \right)=n_{0}^{2}\left( x,y \right)-\Delta n^{2}\sum_{m\neq0} \xi_{m}\left( x,y \right)e^{-{ik}_{g}z} \#(3) \end{aligned}$$

where$n_{0}\left( x,y \right)$ is the unperturbed refractive index, and $\Delta n^{2}$ is the square change in refractive index due to perturbation. The $\xi_{m}\left( x,y \right)$ is the m-th order Fourier coefficient and given by $\xi_{m}\left( x,y \right)=\frac{sin\left( \pi m\mathrm{DC} \right)}{\pi m}$ inside the perturbation region, and 0 everywhere else. Maxwell’s equations for forward propagating mode in unperturbed waveguide become:

$$\begin{aligned} \left( \boldsymbol{\nabla}_{\mathbf{t}}\boldsymbol{+}ik_{g} \right)\boldsymbol{\times}\mathbf{e}_{\mathbf{a}}\left( x,y \right)= i\omega\mu_{0}\mathbf{h}_{\mathbf{a}}\left( x,y \right)\boldsymbol{\#}\left( 4a \right) \end{aligned}$$

$$\begin{aligned} \left( \boldsymbol{\nabla}_{\mathbf{t}}\boldsymbol{+}ik_{g} \right)\boldsymbol{\times}\mathbf{h}_{\mathbf{a}}\left( x,y \right)=-i\omega\epsilon_{0}n_{0}^{2}\left( x,y \right)\mathbf{e}_{\mathbf{a}}\left( x,y \right)\boldsymbol{\#}\left( 4b \right) \end{aligned}$$

When Eqs. (1a)-(1b) are plugged into the Eqs. (2a)-(2b) and fundamental relations of (4a)-(4b) are used, we can obtain the following equations:

$$\begin{aligned} \left( \frac{\Delta\omega}{v_{g}}+i\frac{d}{dz} \right)A+h_{2}B+{ih}_{1t}\left( A+B \right)-{ih}_{1z}\left( A-B \right)=0\#\left( 5a \right) \end{aligned}$$

$$\begin{aligned} \left( \frac{\Delta\omega}{v_{g}}-i\frac{d}{dz} \right)B+h_{2}A+{ih}_{1t}\left( A+B \right)+{ih}_{1z}\left( A-B \right)=0\#\left( 5b \right) \end{aligned}$$

Here, $h_{1t}$ and $h_{1z}$ represent the indirect coupling via radiative mode with transverse and longitudinal field components, while $h_{2}$ is the coefficient of direct coupling. These coefficients can be calculated [1]:

$$\begin{aligned} h_{1t}=\frac{\omega^{4}\Delta n^{4}i}{2k_{\text{g}}c^{4}}\int\int dx dy\int\int dx^{'} dy^{'} \xi_{1}\left( x,y \right)\xi_{1}\left( x^{'},y^{'} \right)\mathbf{e}_{\mathbf{t}}\left( x,y \right){\boldsymbol{\cdot}\mathbf{e}}_{\mathbf{t}}^{\boldsymbol{*}}\left( x^{'},y^{'} \right)G\left( x,x^{'},y,y^{'} \right)\#\left( 6a \right) \end{aligned}$$

$$\begin{aligned} h_{1z}=-\frac{\omega^{4}\Delta n^{4}i}{2k_{\text{g}}c^{4}}\int\int dx dy\int\int dx^{'} dy^{'} \xi_{1}\left( x,y \right)\xi_{1}\left( x^{'},y^{'} \right)e_{\mathbf{z}}\left( x,y \right)e_{z}^{*}\left( x^{'},y^{'} \right)G\left( x,x^{'},y,y^{'} \right)\#\left( 6b \right) \end{aligned}$$

$$\begin{aligned} h_{2}=-\frac{\omega^{2}\Delta n^{2}}{2k_{\text{g}}c^{2}}\int\int(\mathbf{e}_{\mathbf{t}}\left( x,y \right){\boldsymbol{\cdot}\mathbf{e}}_{\mathbf{t}}^{\boldsymbol{*}}\left( x^{'},y^{'} \right)+ e_{\mathbf{z}}\left( x,y \right)e_{z}^{*}\left( x^{'},y^{'} \right))\xi_{2}\left( x,y \right) dx dy\#\left( 6c \right) \end{aligned}$$

where $\mathbf{e}_{\mathbf{t}}\left( x,y \right)$ and $e_{\mathbf{z}}\left( x,y \right)$ are transverse and longitudinal electrical field distributions of unperturbed waveguide. The $\xi_{1}\left( x,y \right)$and $\xi_{2}\left( x,y \right)$ are 1^st^ and 2^nd^ order Fourier coefficients and given by $\xi_{1}\left( x,y \right)=\frac{\sin\left( \pi DC \right)}{\pi}$ and $\xi_{2}\left( x,y \right)=\frac{\sin\left( 2\pi DC \right)}{2\pi}$ (for the rectangular shaped grating) inside the perturbation region and 0 elsewhere. $G\left( x,x^{'},y,y^{'} \right)$ is the Green’s function satisfying:

$$\begin{aligned} \left[ \frac{\partial^{2}}{\partial x^{2}}+\frac{\partial^{2}}{\partial y^{2}}+\frac{\omega^{2}}{c^{2}}n\left( x,y \right)^{2}-k_{\text{g}}^{2} \right]G\left( x,x^{'},y,y^{'} \right)=\delta\left( x-x^{'} \right)\delta\left( y-y^{'} \right)\#(7) \end{aligned}$$

Eqs. (4a)-(4b) can be grouped as follows:

$$\begin{aligned} \frac{d}{dz}\left[ \begin{aligned} A \\ B \end{aligned} \right]=\left[ \begin{matrix} \frac{i\Delta\omega}{v_{g}} & ih_{2} \\ -ih_{2} & -\frac{i\Delta\omega}{v_{g}} \end{matrix} \right]\left[ \begin{aligned} A \\ B \end{aligned} \right]+h_{1t}\left( A+B \right)\left[ \begin{aligned} -1 \\ 1 \end{aligned} \right]+h_{1z}\left( A-B \right)\left[ \begin{aligned} 1 \\ 1 \end{aligned} \right]\#(8) \\ \# \end{aligned}$$

To solve for eigenfrequencies of the system, $A\left( z \right)\sim\exp\left( ikz \right)$ and $B\left( z \right)\sim exp(ikz)$ substitutions are used, where $k$ represents the detuning from the central wavevector $k_{g}$. The resulting dispersion relation is as follows:

$$\begin{aligned} \frac{\Delta\omega_{1,2}}{v_{\text{g}}}=-i{(h}_{1t}-h_{1z})\pm\sqrt{\left( h_{2}+ih_{1} \right)^{2}+k^{2}}\#\left( 9 \right) \end{aligned}$$

For simplicity, we can substitute $h_{1}= h_{1t}+h_{1z}$, and the $\mathrm{Re}\left( h_{2}+ih_{1} \right)=0$ makes the bandgap closure, i.e., EP condition. Since $\xi_{m}\left( x,y \right)$ coefficient is only non-zero in perturbation region and 0 everywhere else, it can be taken out of integration in calculations of coupling coefficients, and Eqs. (6a-6c) can be written as:

$$\begin{aligned} h_{1t}=\frac{\omega^{4}\Delta n^{4}i}{2k_{\text{g}}c^{4}}\left( \frac{\sin\left( \pi DC \right)}{\pi} \right)^{2}\int\int dx dy\int\int dx^{'} dy^{'} \mathbf{e}_{\mathbf{t}}\left( x,y \right){\boldsymbol{\cdot}\mathbf{e}}_{\mathbf{t}}^{\boldsymbol{*}}\left( x^{'},y^{'} \right)G\left( x,x^{'},y,y^{'} \right)\#\left( 10a \right) \end{aligned}$$

$$\begin{aligned} h_{1z}=-\frac{\omega^{4}\Delta n^{4}i}{2k_{\text{g}}c^{4}}\left( \frac{\sin\left( \pi DC \right)}{\pi} \right)^{2}\int\int dx dy\int\int dx^{'} dy^{'} e_{\mathbf{z}}\left( x,y \right)e_{z}^{*}\left( x^{'},y^{'} \right)G\left( x,x^{'},y,y^{'} \right)\#\left( 10b \right) \end{aligned}$$

$$\begin{aligned} h_{2}=-\frac{\omega^{2}\Delta n^{2}}{2k_{\text{g}}c^{2}}\frac{\sin\left( 2\pi DC \right)}{2\pi}\int\int\left( \mathbf{e}_{\mathbf{t}}\left( x,y \right){\boldsymbol{\cdot}\mathbf{e}}_{\mathbf{t}}^{\boldsymbol{*}}\left( x^{'},y^{'} \right)+ e_{\mathbf{z}}\left( x,y \right)e_{z}^{*}\left( x^{'},y^{'} \right) \right) dx dy\#\#\left( 10c \right) \end{aligned}$$

where integrals are carried out over the perturbation region. We notice that both $h_{1}$ and $h_{2}$ are sinusoidal functions of the DC – Duty Cycle, and can be written as $h_{1t}=h_{1t}^{c}\left( \sin\left( \pi DC \right) \right)^{2}$, $h_{1z}=h_{1z}^{c}\left( \sin\left( \pi DC \right) \right)^{2}$, and $h_{2}=h_{2}^{c}\sin\left( 2\pi DC \right),$where $h_{1}^{c}$ and $h_{2}^{c}$ are constant values of these coefficients obtained via integral. The coefficients can be simply plotted as a function of DC:


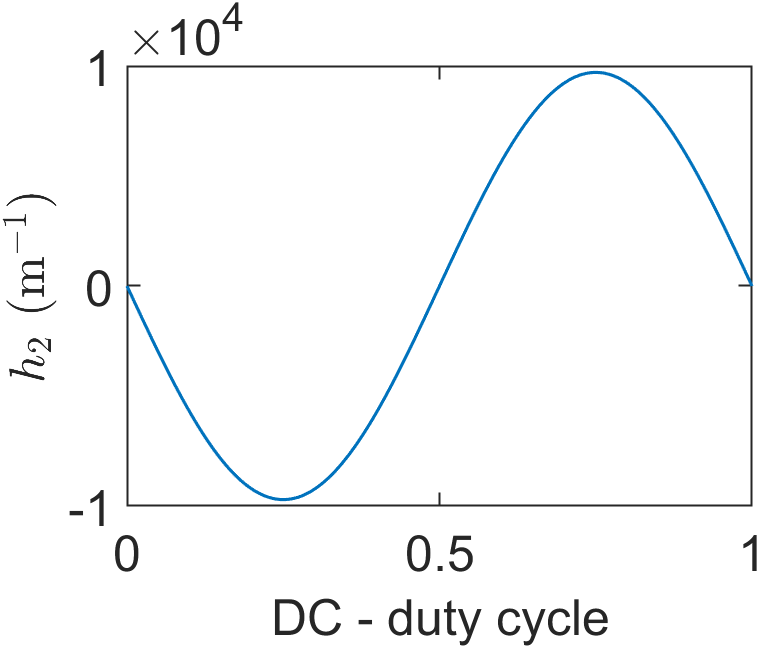

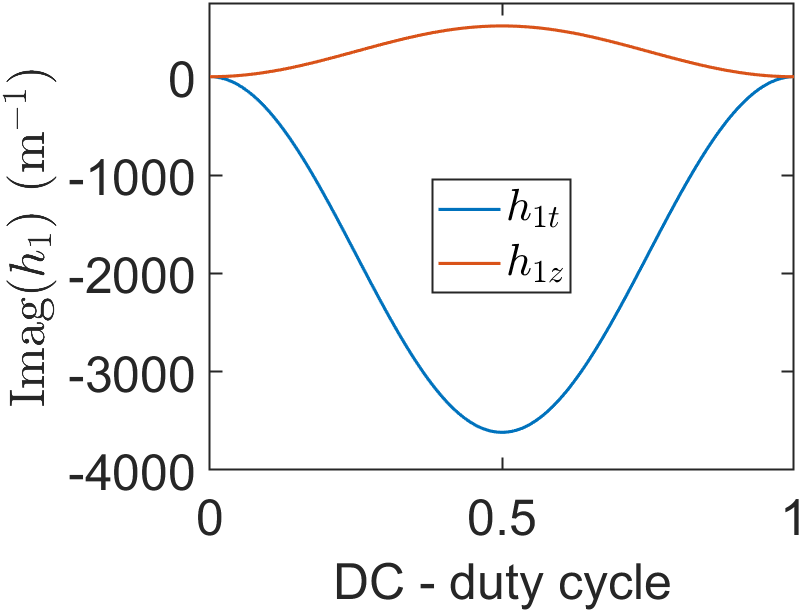

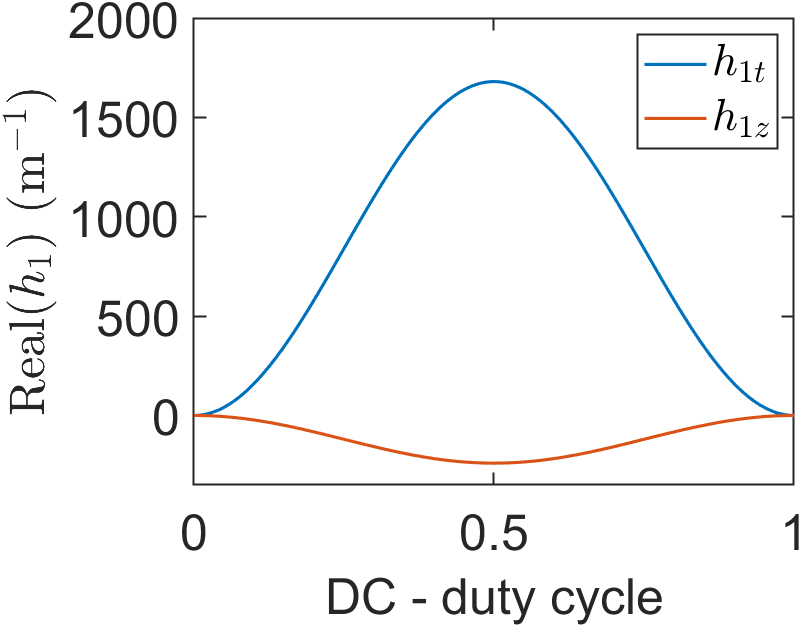


**Fig.S1**. Calculated dependencies of real and imaginary parts of $h_{1}$ and $h_{2}$ on the duty cycle of the grating. Values are calculated by fitting the bandgap curves in Fig.2 of the main article. Both real and imaginary parts of $h_{1t}$ and $h_{1z}$ reach their maximum values when DC = 0.5, while $h_{2}$ crosses zero at this point. As expected, both coefficients vanish for DC = 0 and DC = 1, as these duty cycles correspond to absence of perturbation.

1. **Field profiles inside and outside bandgap closing**


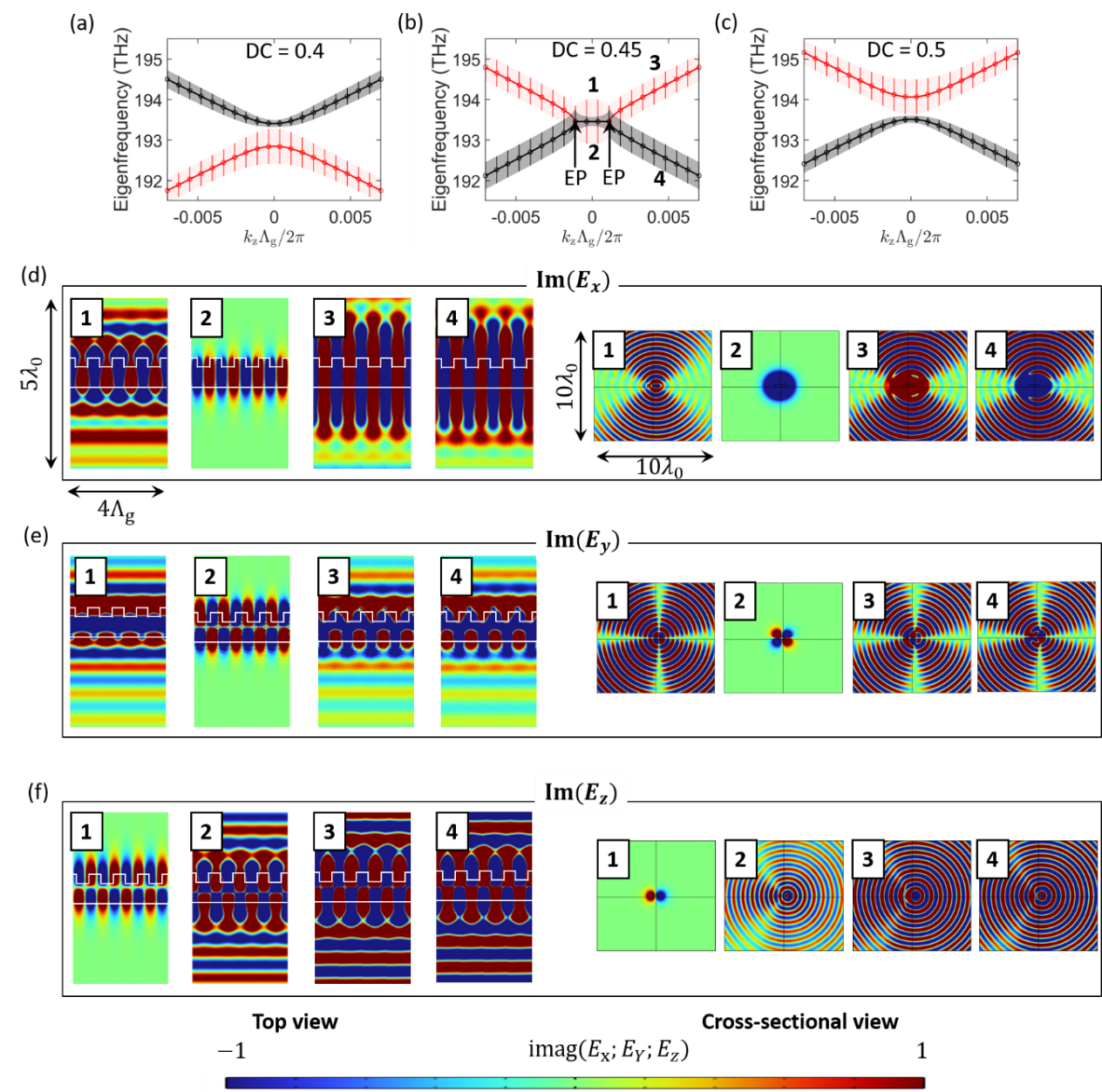


**Fig.S2.** (a-c) Band diagrams for various duty cycles (DCs): (a) 0.4, (b) 0.45, and (c) 0.5, as presented in the paper. (d-f) Field profiles (imaginary $E_{x},E_{y},E_{z}$) of the eigenmodes 1-4 marked in (b). The $E_{y}$ component, similar to $E_{x}$ exhibits symmetric radiative profile for mode 1 and anti-symmetric profile for mode 2, resulting in no radiation. Additionally, outside the bandgap-closing region, plots 3 and 4 indicate nearly equal loss for both modes, reflecting the conventional behavior of a grating coupler.

**3. CMT equation solution for grating modal amplitudes and radiation profile**

To find out the transmission characteristics of the device over finite period of length *L*, we can solve the Eq. (8) with boundary conditions $A \left( z=0 \right)=1$ and $B \left( z=L \right)=0$, and the grating mode profiles are given in the form:

$$\begin{aligned} A\left( z \right)=\frac{ik\cosh\left( ik\left( L-z \right) \right)-\left( i\Delta\beta-h_{1}+2h_{1z} \right)\sinh\left( ik\left( L-z \right) \right)}{ik\cosh\left( ikL \right)-\left( i\Delta\beta-h_{1}+2h_{1z} \right)\sinh\left( ikL \right)}\#\left( 11a \right) \end{aligned}$$

$$\begin{aligned} B\left( z \right)=\frac{i\left( h_{2}+ih_{1} \right)\sinh\left( ik\left( L-z \right) \right)}{ik\cosh\left( ikL \right)-\left( i\Delta\beta-h_{1}+2h_{1z} \right)\sinh\left( ikL \right)}\#\left( 11b \right) \end{aligned}$$

where wavevector $k$ is obtained from Eq. (9):

$$\begin{aligned} k=\pm\sqrt{\left( \Delta\beta+ih_{1}-2ih_{1z} \right)^{2}-\left( h_{2}+ih_{1} \right)^{2}}\#(12) \end{aligned}$$

Transmission at the end of grating $z=L$ is given by

$$\begin{aligned} T=\left| A\left( L \right) \right|^{2}=\left| \frac{ik}{ik\cosh\left( ikL \right)-\left( i\Delta\beta-h_{1}+2h_{1z} \right)\sinh\left( ikL \right)} \right|^{2}\#(13) \end{aligned}$$

Notice that, in the case of $h_{1t}=h_{1z}=0$, corresponding to the absence of radiative coupling, $k=\pm\sqrt{\left( \Delta\beta\right)^{2}-\left( h_{2} \right)^{2}}$ and the transmission equation reduces to:

$$\begin{aligned} T=\left| A\left( L \right) \right|^{2}=\left| \frac{ik}{ik\cosh\left( ikL \right)-i\Delta\beta\sinh\left( ikL \right)} \right|^{2}\#(13') \end{aligned}$$

Equation (13’) represents the standard transmission equation for traditional Bragg gratings, which leads to a simple bandgap in the transmission spectrum. However, our device exhibits Fano lineshapes, characterized by a transmission peak near the bandgap. This distinctive behavior arises from the presence of radiative coupling and the interplay between direct and indirect coupling coefficients. To better understand the effect of radiative coupling (characterized by$h_{1})$ on the bandgap, we plot CMT calculated transmission spectra for different values of $h_{1}$.


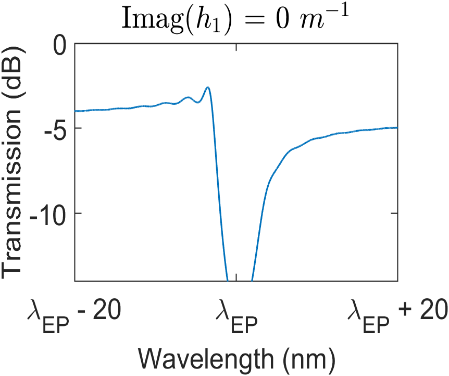

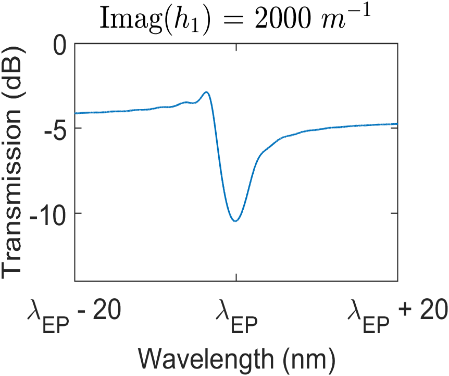

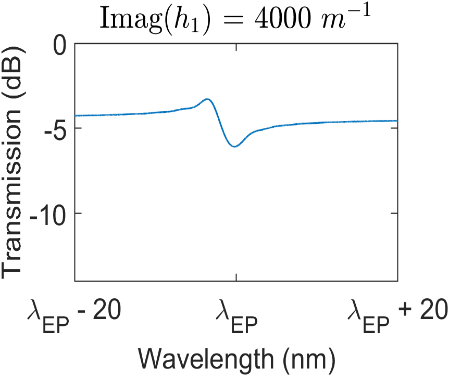

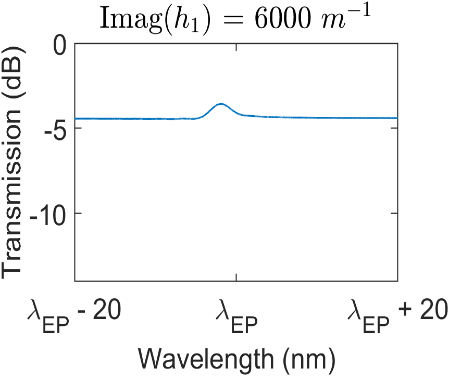

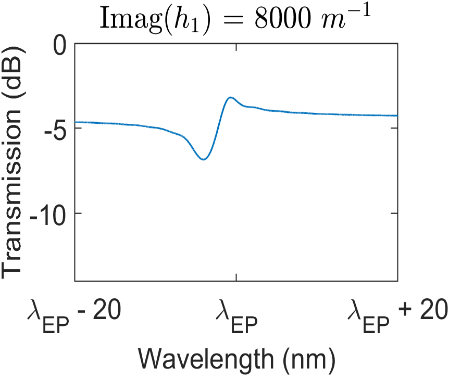

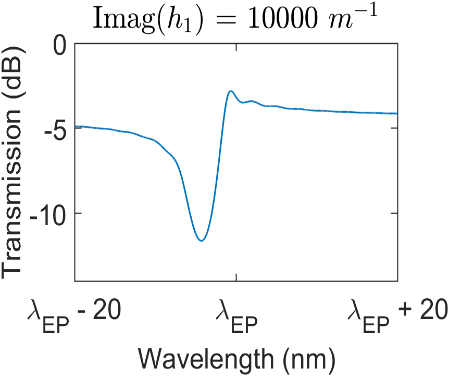


**Fig S3.** CMT calculations of transmission for artificially varied values of $h_{1}$, while $h_{2}$ remains constant. As the figure illustrates, variations in $h_{1}$ lead to a shift in the bandgap and the emergence of a small transmission peak. Notably, at a specific value (6000 m^-1^), the EP condition ${\mathrm{Re}(h}_{2}+ih_{1})=0$ is satisfied, and the bandgap disappears, giving rise to a small transmission peak only. As $h_{1}$ is further increased, the bandgap reopens; however, the bandgap and transmission peak positions are spectrally exchanged, resulting in an inverted lineshape. This phenomenon further highlights the critical role of the radiative coupling coefficient in governing the appearance of inverted Fano lineshapes.

We proceed to investigate the grating mode profiles in the case of $k=0$, which requires $\Delta\beta= h_{2}+2ih_{1z}$, and Eqs. (11a)-(11b) are simplified to linear expressions:

$$\begin{aligned} A\left( z \right)= \frac{ih_{2}-h_{1}}{1-\left( ih_{2}-h_{1} \right)L}z+1\#\left( 14a \right) \end{aligned}$$

$$\begin{aligned} B\left( z \right)=\frac{ih_{2}-h_{1}}{1-\left( ih_{2}-h_{1} \right)L}\left( L-z \right)\#\left( 14b \right) \end{aligned}$$

In EP condition ${\mathrm{Re}(h}_{2}+ih_{1})=0$, Eqs. (14a)-(14b) can be further simplified as:

$$\begin{aligned} A\left( z \right)= -\frac{\mathrm{Re}\left( h_{1} \right)}{1+\mathrm{Re}\left( h_{1} \right)L}z+1\#\left( 14'a \right) \end{aligned}$$

$$\begin{aligned} B\left( z \right)=\frac{\mathrm{Re}\left( h_{1} \right)}{1+\mathrm{Re}\left( h_{1} \right)L}\left( z-L \right)\#(14'b) \end{aligned}$$

The guided power in the waveguide can be expressed as:

$$\begin{aligned} P_{\mathrm{guided}}\left( z \right)=\left| A\left( z \right) \right|^{2}-\left| B\left( z \right) \right|^{2}=A\left( z \right)A^{*}\left( z \right)-B\left( z \right)B^{*}\left( z \right)= \\ =- \frac{2\mathrm{Re}\left( h_{1} \right)}{\left( 1+\mathrm{Re}\left( h_{1} \right)L \right)^{2}}z-\frac{2\mathrm{Re}\left( h_{1} \right)^{2}L^{2}}{\left( 1+\mathrm{Re}\left( h_{1} \right)L \right)^{2}}+1\#\left( 15 \right) \end{aligned}$$

Notice that guided power is a linear function of *z*. This power of propagating modes is lost through radiation, so we can write radiation power as the change rate of guided power:

$$\begin{aligned} P_{\mathrm{rad}}\left( z \right)\propto\left| \frac{d{(\left| A\left( z \right) \right|}^{2}-\left| B\left( z \right) \right|^{2})}{dz} \right|=\frac{2\mathrm{Re}\left( h_{1} \right)}{\left( 1+\mathrm{Re}\left( h_{1} \right)L \right)^{2}}\#\left( 16 \right) \end{aligned}$$

**References**

1. Kazarinov, R. & Henry, C. Second-order distributed feedback lasers with mode selection provided by first-order radiation losses. IEEE Journal of Quantum Electronics 21, 144-150 (1985).

2. Hardy, A., Welch, D. F. & Streifer, W. Analysis of second-order gratings. IEEE Journal of Quantum Electronics 25, 2096-2105 (1989).
